# Modern Medical Rehabilitation Methods for Patients with Peripheral Nerve and Brachial Plexus Injuries (Review)

**DOI:** 10.17691/stm2025.17.2.08

**Published:** 2025-04-30

**Authors:** A.N. Belova, T.S. Kalinina, T.V. Buylova, S.V. Fomin, A.G. Polyakova

**Affiliations:** Professor, Head of the Department of Medical Rehabilitation; Privolzhsky Research Medical University, 10/1 Minin and Pozharsky Square, Nizhny Novgorod, 603005, Russia; Assistant Professor, Department of Medical Rehabilitation; Privolzhsky Research Medical University, 10/1 Minin and Pozharsky Square, Nizhny Novgorod, 603005, Russia; Professor, Department of Medical Rehabilitation; Privolzhsky Research Medical University, 10/1 Minin and Pozharsky Square, Nizhny Novgorod, 603005, Russia; Head of the Department of Medical Rehabilitation and Neurology; National Research Lobachevsky State University of Nizhny Novgorod, 23 Prospekt Gagarina, Nizhny Novgorod, 603022, Russia; Neurologist; Nizhny Novgorod Regional Clinical Hospital named after N.A. Semashko, 190 Rodionova St., Nizhny Novgorod, 603093, Russia; Associate Professor, Department of Medical Rehabilitation; Privolzhsky Research Medical University, 10/1 Minin and Pozharsky Square, Nizhny Novgorod, 603005, Russia

**Keywords:** peripheral nerve injury, brachial plexus injury, medical rehabilitation, physical therapy

## Abstract

Peripheral nerve and brachial plexus injuries represent one of the most serious medical challenges due to the high frequency of disabling consequences. Medical rehabilitation for such injuries is critically important as it ensures the most complete functional recovery for patients.

**The aim of this review** is to summarize and interpret the data on medical rehabilitation methods, as well as to assess the effectiveness of rehabilitation strategies and techniques for restoring upper limb functions after peripheral nerve and brachial plexus injuries.

Information is provided on the theoretical foundations of functional recovery following peripheral nerve and nerve plexus injuries, as well as on factors that may hinder the full functional recovery of patients. There are discussed rehabilitation strategies and methods aimed at accelerating nerve fiber regeneration, preventing complications, correcting cortical plasticity, restoring patients’ functional capabilities, and improving their quality of life. Special attention is given to pain management, electrical stimulation, sensory deficit correction, and physical therapy in the postoperative period.

Rehabilitation modalities and the medical rehabilitation duration are highly individualized and depend on numerous factors that determine the rehabilitation interventions direction. However, a significant number of rehabilitation methods have a low evidence base: many scientific studies are based on small samples, do not consider the heterogeneous nature of injuries, and do not evaluate longterm outcomes. Further research is needed to assess the effectiveness of both individual rehabilitation techniques and comprehensive rehabilitation programs that facilitate the recovery of motor activity in patients with peripheral nerve and brachial plexus injuries.

## Introduction

Peripheral nerve injuries (PNIs) constitute a significant medical issue due to their widespread prevalence and adverse health consequences for patients [1–3]. According to Russian researchers, in the overall structure of peacetime trauma, PNIs occur in 2–6% of cases, with 70% involving the upper extremities, most commonly affecting the median and ulnar nerves [1, 4, 5]. Similar figures are reported in the United States. For instance, a cohort study found that among 1,230,362 individuals with upper and lower extremity traumas, PNIs were diagnosed in 2.6 and 1.2% of patients, respectively [3]. The most severe forms of traumatic peripheral nerve system injuries include brachial plexus injuries (BPIs) [6, 7]. The main causes of traumatic neuropathies in peacetime are traffic accidents, domestic and industrial traumas, while in wartime, battle injuries are the leading cause [1, 3, 8–11]. During military conflicts, the frequency of PNIs can reach 20% of all trauma cases [11]. Military injuries are characterized by the most severe nerve and plexus injuries, often with concomitant damage to bones, vessels, tendons, and muscles [4].

According to research findings [3, 8], PNIs/BPIs predominantly affect young, working-age individuals and can have devastating consequences on patients’ physical and psychological well-being, as well as their socio-economic welfare. For instance, the disability rate associated with PNIs is 60% [11], while that for BPIs is 75% [1]. Medical rehabilitation in cases of PNIs/BPIs is critically important, as it facilitates nerve regeneration (both spontaneous and post-surgical) and ensures the most comprehensive functional recovery [12, 13].

There are significantly fewer publications dedicated to medical rehabilitation in traumatic neuropathies compared to non-traumatic peripheral nerve pathologies [14]. Nevertheless, there have recently appeared new scientific advancements and systematic reviews focusing on rehabilitation aspects of PNIs/BPIs.

**The aim of this review** is to summarize and interpret the data on medical rehabilitation methods, as well as to assess the effectiveness of rehabilitation strategies and techniques for restoring upper limb functions after peripheral nerve and brachial plexus injuries.

## Search strategy and data sources

A systematic literature search was conducted using the following databases and platforms: Scopus, Web of Science, PubMed (MEDLINE and PubMed Central), Springer Link, BioMed Central, Free Medical Journals, SSRN, and Google Scholar. The study used the following key terms: rehabilitation, peripheral nerve, trauma, traumatic peripheral nerve injury, nerve regeneration, brachial plexus, brachial plexus injury, physical therapy, electrical stimulation, therapy modalities, postoperative management.

## Theoretical foundations of functional recovery after peripheral nerve and brachial plexus injuries

Rehabilitative interventions and strategies aim to address the following objectives [10, 15]:

accelerate nerve fiber regeneration during spontaneous recovery or after surgical intervention;
prevent or reduce complications from prolonged immobilization of the injured limb;
correct impaired cortical plasticity, which may impact sensorimotor function recovery due to reduced afferentation;
restore functional capabilities and quality of life in patients.

Rehabilitation specialists should consider casespecific factors that may hinder comprehensive functional recovery.

Contemporary understanding of the pathophysiological mechanisms underlying damaged nerve fiber regeneration is described in various publications in detail [4, 16, 17]. It is worth mentioning that axonal regeneration initiates from the central nerve stump, while the peripheral nerve stump undergoes degeneration, with the intact perineural sheath serving as a conduit for new axon fibers growing towards target organs. Spontaneous recovery of motor function usually reaches a plateau at 18–24 months after PNIs [18].

The severity of PNIs/BPIs complications (e.g., contractures, muscle atrophy, psychological maladaptation) is directly related to the duration of denervation. Cortical plasticity processes can significantly influence functional outcomes after PNIs/BPIs [15, 19]. Denervation of a specific area leads to afferentation disruption, loss of sensory feedback (which, in turn, is accompanied by the reorganization of cortical representations of the denervated organ), as well as decrease in efferent motor signals, alteration of the cortical model of the bilateral somatosensory and supplementary motor cortex, and dysfunctional movements. Axonal sprouting and axon growth misdirection can also result in cortical sensory map disorganization [15, 17].

The primary factors determining the speed and completeness of peripheral nerve fiber regeneration (thus, the scope and focus of rehabilitative interventions) include the severity of neural conductor damage, injury level, nature and duration of the injury, and timely surgical intervention [7, 17, 20, 21].

***The degree of local nerve trunk lesion***, according to the fundamental Seddon classification [22], is determined based on the preservation of the axon and connective tissue structures: neuropraxia refers to nerve damage that does not lead to axonal degeneration; axonotmesis denotes nerve damage resulting in axonal degeneration while preserving the epineurium, perineurium, endoneurium, and Schwann cells; neurotmesis indicates a nerve rupture with the transection of the axon and connective tissue sheaths of the nerve.

A subsequent, more detailed Sunderland classification [23], which identifies five degrees of nerve injury, serves as the basis for predicting the outcomes of spontaneous regeneration [10]. The first degree injury corresponds to neuropraxia and involves demyelination without axon loss; the mechanism of recovery is remyelination; the prognosis is complete spontaneous nerve function recovery for <3 months. The second and the third injury degrees correspond to axonotmesis, with axon damage without or with disorganization of endoneurial architectonics and Wallerian degeneration; recovery mechanisms include axonal sprouting, axon growth (1 mm per day), and muscle fiber hypertrophy. Spontaneous nerve function recovery is partial or complete: prognosis is considered favorable if electroneuromyography demonstrates preservation of impulse conduction along motor/sensory fibers and needle electromyography (EMG) demonstrates only a slight decline in recruitment. The fourth degree injury corresponds to axonotmesis and involves axon damage, massive disorganization of the myelin sheath, endoneurium and perineurium, and the development of Wallerian degeneration; recovery mechanisms include axon growth and muscle fiber hypertrophy; the prognosis is unfavorable and the chance of recovery in the absence of surgical intervention is very low. The fifth degree injury corresponds to neurotmesis, which means that the nerve is severed completely and there is no recovery unless surgical intervention is performed [10].

All of the aforementioned classifications are based on microscopic changes in the nerve trunk. Macroscopically, determining the degree of damage is practically impossible; therefore, diagnosis relies on dynamic clinical observation and electrophysiological studies.

***The level of injury*** (proximal or distal) also significantly impacts the prognosis for spontaneous recovery. The more proximal the lesion of the nerve trunk or plexus (i.e., the greater the distance from the site of injury to the innervated muscle), the poorer the prognosis for functional recovery. This is related to the fact that more time is needed for nerve fiber regrowth and there is a higher probability of developing irreversible scar changes in the endoneural tube of the peripheral nerve segment. For instance, when the axon is damaged at a distance greater than 15–20 cm from the target organ (for example, as in BPI), the spontaneous and complete recovery within 12–18 months is unlikely to happen [10, 24]. In such cases, nerve transfer may become necessary. The worst prognosis is observed in preganglionic injuries, as spontaneous regeneration of spinal nerve roots is impossible, and unique procedures for implanting damaged nerve roots to the spinal cord, performed in some clinics, have yet to find widespread practical application [25].

***The nature of the injury*** also plays a crucial role in predicting nerve function recovery. For example, in acute penetrating lacerations, there is a high probability of complete nerve trunk transection (anatomical interruption). In such situations, the possibility of spontaneous recovery is excluded. In closed traumatic nerve trunk injuries, the preservation of nerve sheaths (axonotmesis) is often observed, which predisposes to the possibility of spontaneous recovery. Conversely, traction injuries frequently damage the central nerve segment, significantly complicating the regeneration process. In gunshot wounds, nerve trunks dysfunction may be attributed to their contusion (neuropraxia), making it prudent to monitor for spontaneous improvement over several weeks after injury, followed by a reassessment of the degree and extent of damage [1, 10].

***The duration since the injury*** has an inversely proportional relationship with the outcome of reinnervation: the longer the time elapsed since the injury, the worse the condition of the target zones, the greater the diastasis between the central and peripheral segments, and the larger the size of the neuroma and fibroschwannoma [4]. Denervated muscles undergo atrophy, and after a certain period, the neuromuscular synapse experiences irreversible changes, making reinnervation impossible [26, 27].

The timeliness and quality of surgical intervention determine the functional outcome in cases where spontaneous nerve regeneration is not anticipated [1, 4, 28]. In the case of a clean stab or laceration wound, when a diagnosis of nerve transection is established, surgery should be performed as early as possible [1, 29]. In gunshot injuries, when there are no obvious signs of anatomical transection, the indications for surgical treatment become apparent no earlier than 2–3 weeks post-injury, when the nerve trunk contusion has significantly regressed [1]. In closed traumatic nerve trunks injuries, the decision regarding surgical intervention is usually made no sooner than 3–4 months post-injury, provided that intensive conservative treatment and dynamic neurophysiological monitoring have been conducted during this time [1, 13]. However, it is unjustifiable to postpone surgical interventions for an extended period, as there exists a finite “window” time period within which a denervated muscle can undergo reinnervation. For instance, nerve transfer surgery, where the distal segment of a transected nerve is attached to an intact donor nerve, should ensure reinnervation of the target muscle within 12–18 months after injury [10], with optimal surgical outcomes achieved within the first 3–6 months [18, 29].

To determine all factors influencing the possibility and degree of spontaneous recovery in PNIs/BPIs, it is essential to conduct a medical history collection, neurological examination, neurophysiological studies (electromyography, needle EMG), and neuroimaging investigations (magnetic resonance neurography, ultrasound) [18, 30]. Thorough patient assessment and diagnostic monitoring enable the evaluation of the trajectory of functional recovery.

## Rehabilitation modalities

The objectives of PNIs/BPIs rehabilitation include stimulating nerve regeneration throughout the duration required for its growth to the target organ; maintaining muscle and joint function; reducing or correcting sensory deficits; providing pain management and psychological support; and restoring patients’ ability to engage in daily and professional activities [12, 31]. To achieve these objectives, the efforts of a multidisciplinary team of physicians are essential, along with the establishment of a rehabilitation diagnosis within the framework of the “International Classification of Functioning, Disability and Health (ICF)”, and the implementation of a wide range of rehabilitation interventions [13, 32].

###  

#### Pain management

Pain may manifest at all PNIs/PBPIs stages (during acute neuromuscular trauma; postoperative pain at the surgical site; chronic neuropathic pain). Significantly hindering active rehabilitation, pain severely deteriorates the mental health and quality of life of the patient. Pain management should begin immediately upon the patient reporting pain [13]. The pain syndrome treatment is conducted according to modern guidelines for neuropathic pain management [33, 34]. This may involve pharmacological agents (antidepressants, anticonvulsants, opioid analgesics), regional anesthesia (interscalene brachial plexus block for BPIs, prolonged interscalene brachial plexus block with perineural catheter insertion with local anesthetic), neurostimulation using implanted electrodes, psychotherapy, and combinations of pharmacological and non-pharmacological treatment methods [33, 34].

Transcutaneous electrical nerve stimulation (TENS) has been reported to be used in PNIs [35]; however, the TENS analgesic effect in traumatic neuropathies, as opposed to tunnel syndromes, remains insufficiently studied [36, 37]. The mechanism of the TENS analgesic effect, based on the “gate control theory of pain”, presupposes the preservation of afferent impulses from the affected limb; therefore, TENS application is not justified in cases of complete nerve trunk transection or preganglionic BPIs [38].

Adjuvant methods for the treatment of chronic neuropathic pain in BPIs include transcranial directcurrent stimulation and repetitive transcranial magnetic stimulation; however, the features of using these methods in PNIs/BPIs require further investigation [39]. Data have been published regarding the effective treatment of chronic neuropathic pain associated with BPIs through invasive deep brain stimulation [40]; however, the insufficient number of studies currently does not allow for reliable conclusions regarding the applicability of this method in PNIs/BPIs [13].

To reduce pain and swelling, as well as prevent the formation of adhesions, some authors recommend low- power laser irradiation (LPLI) [41, 42]. The theoretical basis for LPLI usage lies in its potential ability to reduce the duration of the inflammatory process phases and interstitial tissue edema, as well as to enhance tissue oxygen consumption and blood flow [43–45]. However, the effectiveness of LPLI in treating the upper and lower extremities PNIs has only been demonstrated in non-randomized research with a heterogeneous patient sample [46]. One study reports the effectiveness of hypnotherapy and point massage (acupressure) in treating chronic pain resulting from PNIs [47].

As adjunctive neuropathic pain management, B vitamins in high doses as complexes, as well as alpha- lipoic (thioctic) acid, may be considered [33]. The choice of specific treatment modalities is determined by the severity and localization of the pain syndrome.

#### Neuroprotective pharmacotherapy

The potential for local or systemic application of substances that can enhance regeneration outcomes is actively being studied. These substances aim to influence various factors such as post-traumatic neuronal and glial cell death, proliferation, migration and differentiation of Schwann cells, growth cone motility, and axonal growth orientation [25, 48]. Experiments in animal models [48] and laboratory studies [49] have demonstrated the ability of certain pharmacological agents (e.g., dexamethasone, methylprednisolone, L-carnitine, citicoline, memantine, riluzole, atorvastatin, mesenchymal stem cells, and local application of glial-cell-line-derived neurotrophic factor (GDNF)) to influence Wallerian degeneration, fibrosis, and other processes associated with nerve fiber regeneration. Despite the lack of compelling evidence for the efficacy of these agents in clinical practice, B vitamins and citicoline are utilized as neuroprotectors due to their potential effectiveness, high safety level, and minimal side effects [13].

***Orthotic management*** is used to protect damaged tissues (to prevent uncontrolled movements that may lead to nerve ends separation or damage to sutures/grafts), to prevent or minimize contractures and strains of the tendon-ligamentous apparatus, as well as for physical training (dynamic orthoses) [12, 38, 50–53].

For immobilization purposes, plaster splints and static orthoses are used 2–3 days after the primary surgical dressing removal. The advantages of various types of external fixation have not been studied, thus the selection is based on medical indications and patient tolerance level [13]. The fixation device should be lightweight, not restrict preserved movements, not compress underlying tissues, especially in areas with impaired sensation, and not disrupt limbs circulation. Prior to orthotic management, measures aimed at reducing reactive limb swelling (e.g., retrograde massage, elevated limb positioning) should be implemented [10]. To prevent contractures, the limb segment is typically maintained in a functionally optimal position using an orthosis: in a radial nerve injury, the wrist and fingers are held in an extended position; in a fibular nerve injury, the foot is maintained in a neutral position. For BPIs, the physiological position corresponds to abduction and external rotation of the shoulder, supination of the forearm, and wrist extension. In BPIs with the development of Erb–Duchenne paralysis (shoulder and elbow joints dysfunction), shoulder straps and figure-eight bandages may be used to prevent uncontrolled movements; in cases of Dejerine–Klumpke paralysis (wrist joint dysfunction), a splint or orthosis is utilized to maintain the wrist in an extension position of 10–20°, which serves as a preventing contractures measure and pain reduction [13]. After surgical interventions, during immobilization, the position most favorable for the apposition of the severed nerve ends should be considered.

The duration of immobilization can vary significantly depending on the injury and surgical interventions [12, 52]. Immobilization following nerve suturing typically lasts longer (up to 3 weeks) than for procedures (such as nerve transplantation or repositioning) that do not involve significant nerve tension on the nerve trunks [50]. In some cases, such as finger nerves repair, immobilization may be limited to the initial surgical dressing without subsequent orthotic management [50].

In situations where other soft tissues have been surgically repaired (e.g., tendon repair), the immobilization duration may be extended to ensure sufficient healing of these tissues. For instance, in surgeries involving the brachial plexus and the pectoralis major muscle, immobilization in an adducted and internally rotated shoulder position continues for 4 weeks [50].

As the ability to perform active movements improves, a transition from static orthoses to dynamic orthoses occurs, which are utilized not only during physical training but also to facilitate the performance of daily activities [53].

***Physical therapy*** is a crucial rehabilitation strategy, helping to maintain the contractile muscle properties during denervation and facilitating functional recovery during reinnervation [12, 54]. Targeted physical activity has been shown to enhance intracellular regenerative mechanisms and induce an afferent impulses flow, ensuring appropriate cortical representation of the affected limb [15, 55]. A typical approach, based on the proprioceptive neuromuscular facilitation theory, involves a sequential transition (as muscle strength increases) from passive range of physical exercises to passive exercises with active assistance, then to active exercises in facilitated conditions, and finally to active exercises against gravity and resistance [13, 14]. In BPIs, physical exercises aim not only to restore the range of motion and the affected limb muscle strength but also to train balance, which may be disrupted due to muscle imbalance [12, 56].

Before starting physical therapy, reliable external fixation of the affected limb segments is essential, as well as a preserved motor functions assessment. The duration and intensity of the trainings are quite individual, as the changes in muscle structure and function that determine endurance to physical loads can vary significantly in PNIs. It has been reported that physical therapy based on a person-centered approach is effective even in cases of severe BPIs [12, 57].

***Electrical nerve stimulation*** is prescribed for PNIs/BPIs requiring surgical treatment to accelerate the regeneration of damaged nerve fibers [27, 58–60]. The molecular mechanisms of peripheral nerve regeneration under direct electrical stimulation have been studied in animal models for several decades. It has now been established that electrical stimulation can affect neurons and activate intracellular regeneration mechanisms, stimulating the synthesis of proteins necessary for axon growth and sprouting [27, 61, 62]. It is believed that electrical stimulation effects are mediated by secondary messengers released in response to stimulation, which activate ATP-mediated molecular pathways of regeneration [27]. Numerous studies on rodents have confirmed the high potential of electrical stimulation in various types of PNIs, including compression, transection, and extensive defects [29, 63, 64]. It has also been demonstrated that applying electrical stimulation to a healthy nerve prior to injury (nerve “preconditioning”) promotes accelerated regeneration after trauma [65, 66].

The results of animal experiments have led to the clinical application of direct electrical nerve stimulation, either intraoperatively or through electrode implantation [67–71]. Direct electrical stimulation can be performed during surgery or in the postoperative period. Randomized clinical trials have confirmed that intraoperative electrical nerve stimulation according to a standard protocol (frequency 20 Hz, stimulation duration 1 h) accelerated recovery and improved treatment outcomes for patients with PNIs [67–69, 71]. A detailed description of the intraoperative electrical stimulation parameters is provided in the review by Juckett et al. [27]. Another study [72] demonstrates the effectiveness of direct electrical nerve stimulation with low-intensity alternating current (frequency 8 Hz, amplitude 20– 40 mA). The procedure was performed postoperatively daily for 14 days, twice a day for 15 min, using electrodes implanted during bone osteosynthesis.

In the systematic review by Costello et al. [59], the effectiveness of electrical stimulation was analyzed in 229 patients with BPIs and upper extremity nerve injuries. The protocols of the analyzed studies varied: one session of the intraoperative electrical stimulation, implantation of electrodes with stimulation commencing immediately after nerve suturing, and TENS in cases of unoperated nerve compression injuries. The duration of the sessions ranged from 20 min to 1 h, with a stimulation frequency of 20 Hz, intensity varying from 3 to 30 V, and pulse durations ranging from 0.1 to 1.0 ms. Patients were monitored for an average of 13.5 months. Despite significant differences in the study protocols, functional outcomes for patients receiving electrical stimulation were significantly better than those in the control groups. It is noteworthy that among the six randomized studies analyzed in the review, only two dealt with traumatic nerve injuries (transection and iatrogenic traction), while the others involved stimulation for chronic compression injuries with pain syndrome.

Direct electrical nerve stimulation should be combined with physical therapy, as these two rehabilitation methods complement each other and may have a synergistic effect on regeneration [26, 54, 73–75].

The question of the clinical utility of electrical stimulation for preserving denervated muscles remains contentious [50, 76, 77]. It is known that this method is successfully applied to increase strength and improve the function of weak muscles with intact innervation [78]. However, there is very little scientific research dedicated to the application of electrical stimulation to preserve the function of muscles denervated due to PNIs or muscles awaiting reinnervation, and the results of these studies are contradictory [50, 76]. It is essential to differentiate between the state of muscle denervation resulting from lesions of upper (central) and lower (peripheral) motor neurons. With an intact lower motor neuron, electrical stimulation induces muscle contraction through the depolarization of the peripheral nerve, as the excitation threshold of the neurolemma is lower than that of the sarcolemma. In cases of lower motor neuron damage, electrical stimulation may induce muscle contraction only through direct muscle fibers stimulation and sarcolemma depolarization. The stimulus duration required for muscle contraction in a lower motor neuron injury often exceeds 1 ms, and the current intensity required for sarcolemma depolarization is significantly higher than that for the neurolemma. Electrical stimulation with such parameters is often painful, which limits the method’s applicability [78]. Furthermore, it remains unclear how electrical stimulation affects nerve regeneration in denervated muscles and whether it inhibits this process.

In experiments conducted on rats, it was demonstrated that direct electrical stimulation of denervated skeletal muscles exacerbated their atrophy and impaired the neuromuscular functions recovery [79]. In another experiment [80], the effects of high-frequency (100 pulses per second, pulse duration of 80 μs) and low-frequency (4 pulses per second, pulse duration of 240 μs) TENS on the sciatic nerve fibers regeneration in mice following crush lesion were compared. It was shown that TENS resulted in axonal swelling and the nerve fibers cytoarchitecture disruption, with changes being more significant in the group of mice subjected to high- frequency stimulation.

The systematic review of publications [76] devoted to the electrical stimulation efficacy in patients with neurological disorders did not yield definitive conclusions either in favor of or against the use of this method for increasing muscle strength after PNIs. Overall, to expand the clinical application of electrical stimulation in cases of PNIs, larger-scale clinical studies involving homogeneous patient groups are required, as well as the development of commercially available devices for conducting stimulation [10].

#### Sensory deficit correction

Sensory disorders have a detrimental impact on an individual’s quality of life, particularly when affecting the hands. Initially, patients with sensory deficits are taught protective and compensatory strategies: they are warned about the burns and frostbite risks for impaired sensitivity areas, informed about the necessity of wearing gloves or mittens in cold environments, and cautioned against contact with heated objects and hot water, among other precautions [10]. In cases of hypersensitivity and allodynia, the consideration of recognized nonpharmacological and pharmacological strategies should be explored [15, 81].

Due to the sensory brain map disorganization, PNIs/BPIs patients who have undergone nerve transfer surgeries may experience referred sensations in unaffected body parts and distorted perception of sensations in the affected limb [15, 82, 83]. To correct sensory deficits, sensory relearning techniques based on the cortical remodeling theory are recommended [15, 83, 84]. A classic method of sensory retraining involves tactile gnosis relearning through interaction with objects of various textures, temperatures, shapes, and volumes [84]. The traditional method of “sensory reeducation” has shown effectiveness as a component of comprehensive medical rehabilitation; however, clinical studies validating its efficacy with sufficient sample sizes have not been conducted [13].

Classic techniques also include activity-based sensory reeducation [15] implemented during occupational therapy. Additionally, patients are encouraged to utilize hand function in their daily professional activities (if there are no contraindications) [84].

To correct sensory deficits in the hand, the “mirror therapy” method is used. This technique is based on visual feedback: a mirror is positioned in front of the patient in a way that allows them to see the reflection of their healthy hand, with the movements of that hand perceived as movements of the affected hand. It is believed that “mirror therapy” stimulates cortical areas that do not receive afferent signals from the affected hand, helping to restore functional connections between the limb and the brain’s cortex [15, 31]. Some studies suggest that this therapy may surpass traditional sensory reeducation methods [31]. The effectiveness of mirror therapy may be enhanced when combined with transcranial direct current stimulation [85].

A modern strategy for sensory relearning is the technology of cross-modal sensory substitution, where tactile information is transformed into other sensory modalities, such as visual or auditory signals, using high-tech devices (like sensory gloves). As a result of training, neural connections between sensory cortex areas begin to process auditory or optical signals as tactile sensations, creating the illusion of afferentation from the affected limb [13, 15].

#### Occupational therapy

It has been proven that meaningless imitative exercises are less effective than purposeful activities, which initially concerns the upper limb [86, 87]. Therefore, a mandatory rehabilitation component for PNIs/BPIs patients is doing targeted exercises to enhance the patient’s ability to adapt to daily life. For instance, a prospective study [87] demonstrated that regularly practiced activities such as folding a towel, lifting a bag, using a knife for cutting, drinking water from a glass, and using utensils accelerated the functional recovery of patients who underwent surgery for BPIs.

Reflex therapy is applied for PNIs/BPIs not only in traditional Chinese medicine but also in other countries, owing to the phenomenon of neuroplasticity [88]. This ability stimulates the recovery and regeneration of damaged nerves, enhances local blood flow in the affected area, and reduces postoperative pain [89]. There are some publications discussing the theoretical justification for this method in PNIs; for example, a study on animals showed that electroacupuncture could protect from brachial plexus damage by slowing the degeneration of damaged neurons [90]. In a placebo- controlled study on animals experiencing acute phases of experimental pain syndrome caused by double ligation of the sciatic nerve, positive effects of laser acupuncture were observed on the stimulation of key systemic protective responses, including antioxidant defense, autonomic, and microcirculatory indicators [91].

Acupuncture has been shown to be effective in neurological deficits caused by PNIs, with potential mechanisms responsible for its effects including the remodeling of the nervous system during nerve recovery [92]. Yang et al. [93] believe that acupuncture promotes nerve regeneration and axonal growth by activating retrograde transport of related neurotrophins, such as nerve growth factor (NGF), brain-derived neurotrophic factor (BDNF), GDNF, N-cadherin, and microRNA. The feasibility of combining reflex therapy with other treatment methods is also highlighted.

#### Psychosocial support

Emotional and social factors can significantly influence PNIs/BPIs rehabilitation, particularly among young working-age men [13]. Patients often struggle to accept a long recovery period and the possibility of disability, which can lead to depression and maladaptive behaviors, especially several months after the injury. Therefore, psychological assistance — including psychotherapy, medication management, and family involvement — is an essential medical rehabilitation component. It is important for psychologists to help patients develop adaptive psychological strategies that foster active positive awareness and reduce distress levels [94].

***The perspectives of rehabilitation support*** for patients with PNIs/BPIs is linked to advancements in surgical interventions for extensive and proximal PNIs, the use of nanoparticle therapies and neurotrophic factors, and the development of artificial intelligence, robotics, and virtual reality technologies, along with improvements in dynamic orthoses [25, 53, 95–98]. The best outcomes are achieved through the comprehensive application of individually tailored rehabilitation methods [56, 99].

## Physical therapy peculiarities in the postoperative period

The primary objectives of medical rehabilitation in the postoperative period include the elimination or reduction of postoperative edema and pain syndrome; acceleration of nerve regeneration and prevention of excessive scarring at the site of sutures/anastomoses or neurolysis of nerve trunks; prevention of joint contractures and muscle atrophy; compensation for lost movements through synergistic muscles that retain innervation; psychotherapeutic intervention for the patient; and facilitation of functional recovery. Overall, randomized controlled trials recommend the use of rehabilitation techniques and technologies that are not significantly different from those applied during spontaneous recovery; however, physical therapy may have specific characteristics related to the nature of the surgical intervention [100, 101].

There is a general consensus on the necessity of early initiation of passive exercises aimed at preserving the mobility of proximal and distal joints and local immobilization of the nerve suture/anastomosis site during the acute postoperative period [13]. However, it is crucial to protect the surgical site. It is extremely important to have the suture/anastomosis site tensionfree, especially after nerve restoration with significant local tension. The postoperative immobilization duration is quite individual, and there are no universally accepted timelines for immobilization. When addressing issues related to immobilization and the expansion of motor activity, it is advisable to refer to the 5 motor rehabilitation phases proposed for patients who have undergone nerve transfer surgery [51, 102]. These phases are based on the nerve conduction recovery stages and can serve as the foundation for rehabilitation strategies after nerve suturing or autoplasty.

The first phase is from 0 to 3 weeks after surgery. The objective of medical rehabilitation is to protect the apposed nerve ends and control edema. It is crucial to avoid excessive tension on the apposed nerve segments during the patient’s movements. To ensure minimal tension at the suture sites, orthotic devices are utilized [50]. To maintain mobility, gentle exercises are employed to preserve the range of motion in non-adjacent joints. To reduce edema associated with prolonged immobilization, retrograde massage, compression stockings, and proper positioning are recommended.

The second phase is the period between the third week after surgery and early reinnervation, referred to as the “silent” phase due to the absence of active movements [102]. Signs of early reinervation include polyphasic potentials of newly forming motor units detected via needle EMG, as well as muscle contractions that can be palpated during active movement attempts [10]. Muscle strength, assessed using the Medical Research Council (MRC) scale [103], is rated at 0–1. The rehabilitation goal during this phase is to gently correct motor impairments. Passive motion range is maintained, orthotic devices are used to prevent contractures, and stimulation of the function of intact muscles is performed, along with ideomotor exercises (mental visualization of movements without actual execution) to support cortical plasticity processes [31].

The third phase extends from the moment palpable contractions of muscle fibers are observed (MRC — score 1) until the patient begins to demonstrate active movements in positions that exclude the effects of gravity (MRC — score 2). Active movements are trained while eliminating gravitational forces, utilizing suspension systems or dynamic orthoses to facilitate functional movement patterns.

For patients who have undergone nerve transfer surgery, “motor relearning” technologies, such as the donor activation focused rehabilitation approach (DAFRA), are considered effective [101]. This approach is based on the anatomical aspects of the surgical intervention and aims to assist the patient in independently reproducing new target movements [101]. During “donor activation”, the patient activates the original target muscle of the donor nerve to elicit movement in the recipient muscle. For instance, in the case of reinnervating the deltoid muscle using a donor fascicle from the median nerve, which innervates the m. flexor digitorum superficialis, elbow flexion may be initiated by flexing the fingers [104]. Motor retraining is further enhanced through the use of biofeedback technologies based on EMG signals and “mirror” therapy [31, 104].

The fourth phase begins when patients are capable of performing active movements that overcome the force of gravity or light resistance (MRC — score 2–3). The focus of rehabilitation activities is to increase muscle strength through exercises that progressively enhance the amplitude of active movements (physical exercises, electrical stimulation). The range of functional movements is also expanded. An effective method of rehabilitation for patients who have undergone nerve transfer surgery may include EMG-triggered electrical stimulation, which facilitates the association between the activation of the donor nerve and the recipient muscle [102, 105].

The fifth phase corresponds to the period when the patient is capable of performing movements with full amplitude while overcoming the force of gravity (MRC >3). Rehabilitation protocols involve resistance training exercises, electrical stimulation, and training in the performance of routine daily or occupational activities.

## Conclusion

The rehabilitation modalities and the medical rehabilitation duration for peripheral nerve injuries and brachial plexus injuries are highly individualized and depend on numerous factors that influence the rehabilitative interventions directions. A significant number of methods exhibit a low evidence level; many scientific studies are based on small sample sizes, do not account for the heterogeneous nature of the injuries, and fail to evaluate long-term outcomes. Further research is required to assess the effectiveness of both separate rehabilitation technologies and comprehensive rehabilitation programs that facilitate the motor function restoration in patients with peripheral nerve and brachial plexus injuries, enabling their return to normal life. Future prospects for rehabilitative care for these patients are associated with the use of nanoparticle therapy and neurotrophic factors, as well as advancements in artificial intelligence, robotics, and virtual reality.
